# Recurrent uterine tumors resembling ovarian sex-cord tumors with the growth regulation by estrogen in breast cancer 1-nuclear receptor coactivator 2 fusion gene: a case report and literature review

**DOI:** 10.1186/s13000-020-01025-8

**Published:** 2020-09-14

**Authors:** Bin Chang, Qianming Bai, Lin Liang, Huijuan Ge, Qianlan Yao

**Affiliations:** 1 Department of Pathology, Fudan University Shanghai Cancer Center, Shanghai, China; 2 Department of Oncology, Shanghai Medical College, Fudan University, Shanghai, China

**Keywords:** Uterine tumor resembling ovarian sex-cord tumor, Growth regulation by estrogen in breast cancer 1-nuclear receptor coactivator 2 fusion gene, Growth regulation by estrogen in breast cancer 1-rearrangement, Recurrence, Case report

## Abstract

**Background:**

Uterine tumors resembling ovarian sex-cord tumors (UTROSCTs) are rare mesenchymal neoplasms predominantly arising in perimenopausal and postmenopausal women. UTROSCTs with growth regulation by estrogen in breast cancer 1 (*GREB1*)-rearrangement or *GREB1*-rearranged uterine tumors are exceptionally rare, with only 12 previously reported cases. Here, we report a case of UTROSCT with the *GREB1*-nuclear receptor coactivator 2 (*NCOA2*) fusion gene.

**Case presentation:**

A 57-year-old woman presented with a 10.0 cm uterine mass. The tumor was composed of short spindle or epithelioid cells, arranged in diffused sheets, nested, and trabecular/cordlike. The tumor harbored the *GREB1-NCOA2* fusion gene, as confirmed by RNA sequencing. The tumor recurred in the pelvis at 30 months after the initial diagnosis. We also compared the clinical and pathologic features of this case with those of the 12 previously published uterine *GREB1*-rearranged tumors. Of the combined 13 cases (present case and 12 previous cases), the mean age of patients was 64.8 years (range, 51–74 years). Of the nine reported cases of *GREB1*-rearranged tumor with follow up, four cases recurred or metastasized (44.4%). Microscopically, most tumors (10/12, 83.3%) showed infiltrative growth, and two were well demarcated. Mitotic figures ranged from 0 to 14 per 10 high-power fields (2 mm^2^; mean: 3.6). Lymphovascular invasion and necrosis were each present in two cases (2/12, 16.7% and 2/7, 28.6%, respectively).

**Conclusions:**

This case provided further evidence that UTROSCTs with *GREB1*-rearrangement may have a high risk of recurrence/metastasis. Further studies are necessary to clarify the clinical features of this type of tumor, particularly the prognosis, potential treatment, and range of possible molecular events.

## Introduction

Uterine tumors resembling ovarian sex-cord tumors (UTROSCTs) are rare mesenchymal neoplasms that predominantly arise in perimenopausal and postmenopausal women. Recently, new fusion genes were identified in UTROSCTs, including estrogen receptor 1 (*ESR1*)-nuclear receptor coactivator (*NCOA3*), *ESR1-NCOA2*, growth regulation by estrogen in breast cancer 1 (*GREB1*)-*NCOA1*, *GREB1-NCOA2*, *GREB1*-catenin beta 1 (*CTNNB1*), *GREB1*-nuclear receptor subfamily 4 group A member 3 (*NR4A3*), and *GREB1*-synovial sarcoma translocation, chromosome 18 (*SS18*) [1–4].

Clinically, UTROSCT is regarded as a tumor of low malignant potential (5.9% recurrence rate) [5]. However, a recent large series reported that 23.5% of UTROSCT behaved in a malignant manner [6]. Due to its rarity and the limited follow-up information for available cases, the intrinsic molecular mechanisms of UTROSCT cases with different clinical behaviors are still unclear. Lee et al. found that *GREB1*-rearranged uterine tumors may have a higher tendency of aggressive behaviors [4].

Herein, we report a case of recurrent UTROSCT with the *GREB1-NCOA2* fusion gene, summarize the cases reported to date, and discuss the clinical treatment options.

## Case presentation

A 57-year-old postmenopausal woman presented with a 10.0 cm uterine mass, which was suspected to be uterine leiomyoma on B-scan ultrasonography. The mass was identified on physical examination, and there were no clinical manifestations. The patient underwent a total hysterectomy with bilateral salpingo-oophorectomy. Gross examination showed an intramural, 10.0 × 8.5 × 8.0 cm, yellow, greyish white, soft and fleshy, well-circumscribed mass. Both ovaries and fallopian tubes were grossly normal.

**Fig. 1:**
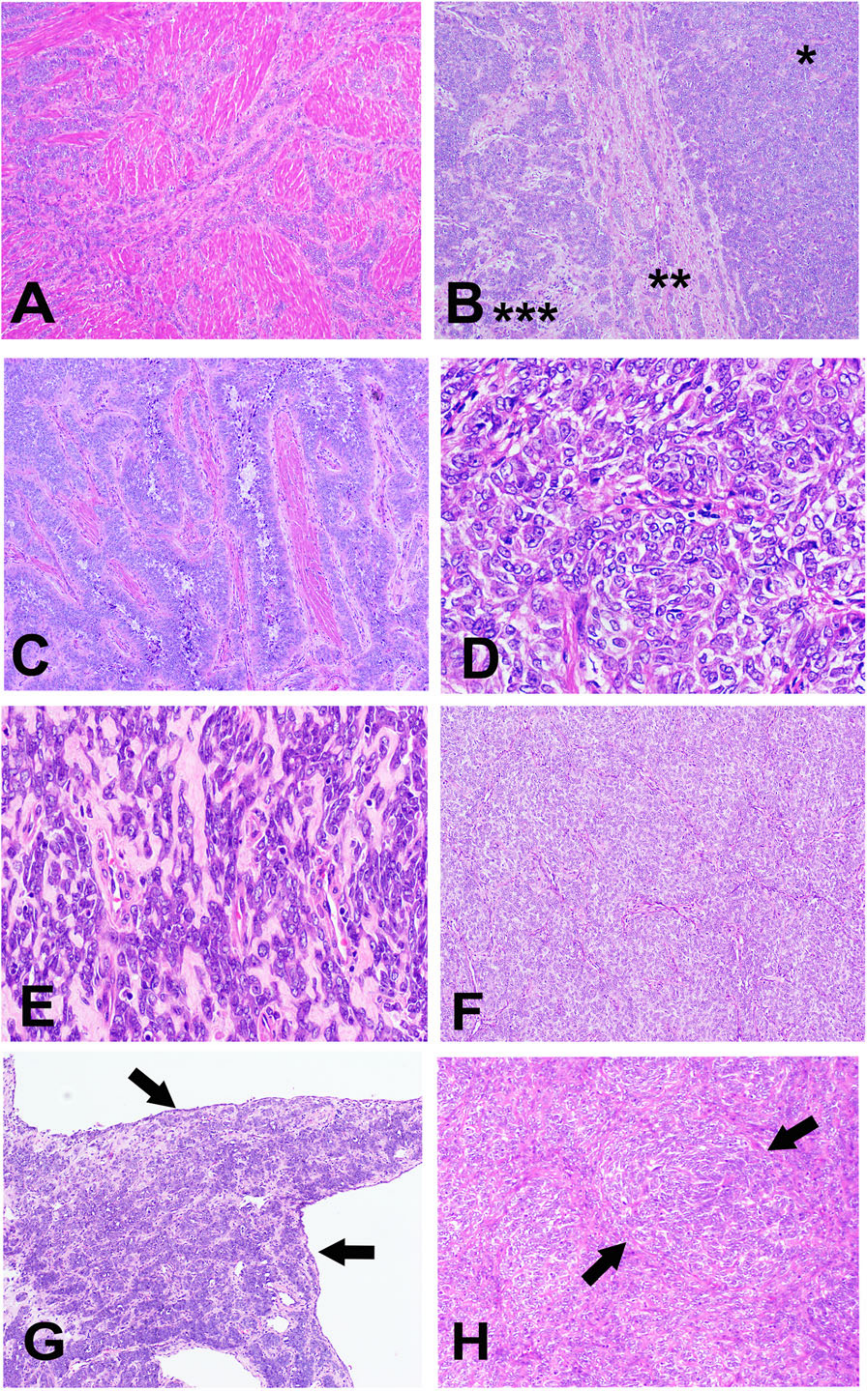
Histological and cytological features of UTROSCT. **a** The tumor showed infiltrative growth into the myometrium (× 20). **b** The tumor contained diffuse sheets (*), trabecular/cords (**), and nested growth patterns (***) (hematoxylin and eosin stain, × 100). **c** Focal anastomotic glandular growth pattern (× 200). **d** The tumor was composed of short spindle and epithelioid cells with scant eosinophilic cytoplasm and round to ovoid bland vesicular nuclei with small or medium prominent nucleoli (× 400). **e** The tumor contained thin-wall vessels in the stroma, and hyaline degenerated fibrous stroma was observed in the focal area (× 400). **f** The recurrent tumor was composed of short spindle and epithelioid tumor cells arranged in inconspicuous nodules with few stroma (× 200). **g** Cordlike pattern and the tumor with cystic changes (× 100). **h** Focal whorls were present in the recurrent tumor (× 200)

Histologically, the mass exhibited myometrial invasion with multiple nodules and irregular cords (Fig. 1a), and the tumor was arranged in diffused sheets mimicking low-grade endometrial stromal sarcoma (45%), nested (30%), trabecular/cordlike (20%; Fig. 1b). Additionally, the mass showed anastomotic glandular growth patterns (5%; Fig. 1c). The tumor was composed of short spindle or epithelioid cells with scant eosinophilic cytoplasm and round to ovoid bland vesicular nuclei with one to two small distinct nucleoli (Fig. 1d). The nuclei were minimally atypical, and the mitotic rate was approximately 3 mitotic figures per 10 high-powered fields. The tumor contained thin-wall vessels in the stroma and hyaline degenerated fibrous stroma was observed in the focal area (Fig. 1e). Necrosis was absent.

**Fig. 2:**
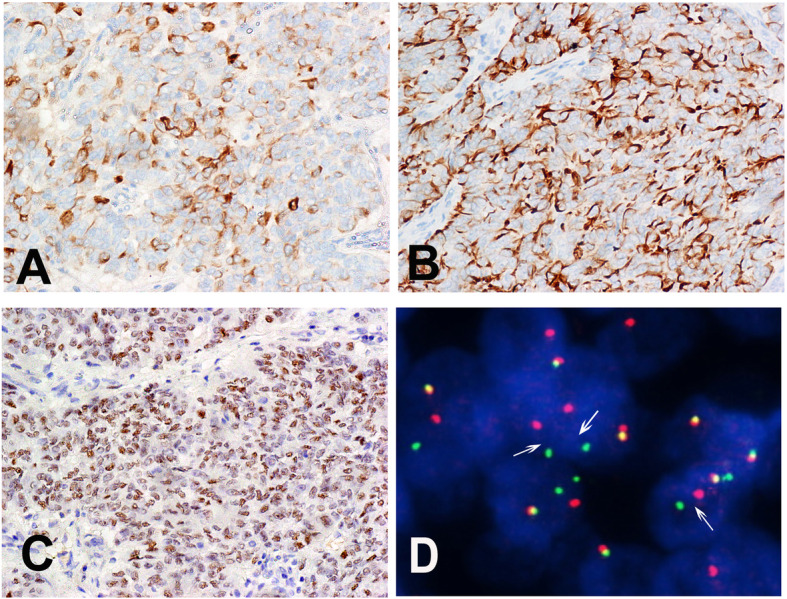
Immunohistological and molecular features of the UTROSCT. **a** AE1/AE3 was positive in the UTROSCT (× 400). **b** Neoplastic cells expressed desmin in the cytoplasm (× 400). **c** Neoplastic cells expressed WT1 in the nuclei (× 200). **d** FISH studies. NCOA2 showing split-apart red and green signals in the tumor cells

Immunobiologically, the tumor diffusely expressed estrogen receptor (ER), progesterone receptor (PR), AE1/AE3 (Fig. 2a), desmin (Fig. 2b), Wilm’s tumor-1 (WT-1; Fig. 2c), CD56, and CD99. Epithelial membrane antigen (EMA) and β-catenin were focally membrane positive. Calretinin, α-inhibin, FOXL2, stromal factor 1 (SF1), CD10, smooth muscle actin, h-Caldesmon, paired box gene 8, cytokeratin 7, cyclin D1, high-mobility group box 45 (HBM45), and Melan-A (A103) were all negative. The Ki67 index was 5%. UTROSCT was diagnosed based on the morphological features and immunophenotype. The patient did not receive any chemotherapy or radiotherapy.

Thirty months later, the tumor recurred with a 7.0 × 6.0 × 5.0 cm pelvic nodule. The patient underwent a pelvic mass resection. Morphologically, the recurrent tumor was similar to the original specimen. Epithelioid or short spindle tumor cells were arranged in nested (80%; Fig. 1f), cordlike (15%; Fig. 1g), and focal whorl (5%; Fig. 1h) patterns. Cystic changes were observed in the focal area (Fig. 1g). The immunophenotype was virtually identical to that of the original neoplasm.

**Fig. 3:**
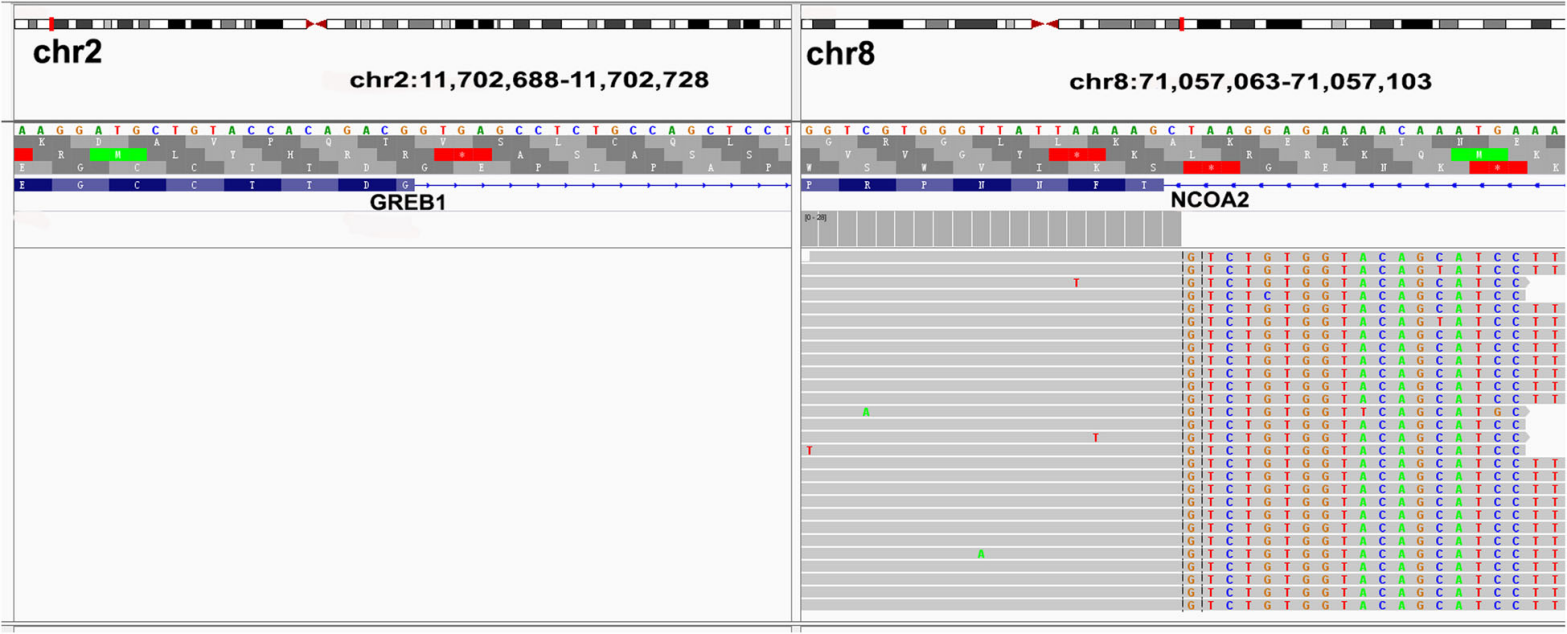
Detailed RNA sequencing of fusion junction reads confirmed the presence of the *GREB1-NCOA2* fusion gene

Molecularly, the break apart fluorescence in situ hybridization (FISH) [7] for *NCOA2* (Abbott Molecular, Abbott Park, IL, USA) revealed that more than 50% of the recurrent tumor cells showed a separated red and green signal probe that was consistent with a chromosome translocation involving the *NCOA2* gene (Fig. 2d). RNA sequencing (NextSeq Reagent Kit, Illumina, Inc., San Diego, CA, USA) using RNA from formalin-fixed, paraffin-embedded specimens [8] revealed an in-frame gene fusion between *GREB1* exon 3 (NM_014668.3) and *NCOA2* exon (NM_006540.3) (t (2;8)(p25;q13) (Fig. 3). Slides of primary and recurrent neoplasm samples were reviewed by two gynecological pathologists (BC and HJG), and the diagnosis of UTROSCT was confirmed. Following pelvic mass resection, the patient received chemotherapy with 3 cycles of paclitaxel liposome and carboplatin and showed no evidence of lesions for 5 months after surgery in the last follow-up.

## Discussion

UTROSCTs are rare neoplasms of unclear histogenesis that are typically located in the uterine corpus and rarely in the endocervix. The morphological features of UTROSCT overlap with those of ovarian sex-cord tumors. The World Health Organization classifies UTROSCTs in the category of “endometrial stromal and related tumors” [9]. However, genetically, these tumors are separate entities that harbor different molecular abnormalities and have different prognosis. The majority of low-grade endometrial stromal sarcomas (LG-ESSs) harbor a fusion gene, most commonly involving *JAZF1*, *SUZ12*, and/or *PHF1* genes, i.e., *JAZF1*-*SUZ12* fusion (80%), *JAZF1-PHF1* fusion (6%), or *EPC1-PHF1* fusion (4%) [10–12]. The rare fusions of *MEAF6-PHF1* (3%) and *MBTD1-CXORF67* (2%) have also been reported in LG-ESSs [12–14]. In contrast, high-grade endometrial stromal sarcomas (HG-ESSs) have been found to harbor *YWHAE-NUTM2A/B* or *ZC3H7B-BCOR* fusion genes and to exhibit more aggressive behavior [15–18].

Recently, characteristic fusions involving *ESR1* and *GREB1*, which are key factors in sex hormone pathways, were identified in 36 cases of UTROSCT; these included *ESR1-NCOA3* (*N* = 15), *ESR1-NCOA2* (*N* = 8), *GREB1-NCOA1* (*N* = 5), *GREB1-NCOA2* (*N* = 4), *GREB1-CTNNB1* (*N* = 1), *GREB1-NR4A3* (*N* = 1), *GREB1-SS18* (*N* = 1), and NCOA2 rearrangement with an unknown partner gene (*N* = 1) [1–4, 19, 20]. Although most cases exhibit benign behavior, UTROSCT is considered to have uncertain malignant potential owing to its low rate of recurrence (5.9%) [5]. However, a recent large series reported that up to 23.5% of UTROSCT cases develop extra-uterine recurrence [6]. The intrinsic molecular mechanisms of UTROSCT that underly its different clinical behaviors are still unclear. In the current case of recurrent UTROSCT in a postmenopausal woman with *GREB1-NCOA2* fusion gene, it is possible that the *GREB1* rearrangement contributed to the aggressive behavior.

The clinical symptoms of UTROSCT are not specific. Typically, UTROSCTs are well circumscribed but unencapsulated. True myometrium invasion is uncommon but can be present in some cases and may be correlated with aggressive behavior. The tumor cells are organized in sheets, nests, trabeculae, or hollow or solid tubules with a repetitive pattern of cordlike or tubular growth; more rarely, the cells will have retiform or glomeruloid appearance or papillae and a solid pattern predominance. Neoplastic cells are small, round, ovoid to spindle with monotonous nuclei, inconspicuous or small to medium with distinct nucleoli, and occasionally have vascular invasion, heterologous elements, or necrosis. Recently, three cases of UTROSCTs with extensive rhabdoid differentiation and malignant behavior have been reported [20]. UTROSCTs exhibit a co-expression of epithelial, smooth muscle, sex-cord markers, and steroid receptors.

*GREB1*-rearranged uterine tumors often show prominently fascicular spindle cells or trabecular/cord-like arranged epithelioid cells and an inconspicuous immunophenotype of sex-cord differentiation [4]. Similarly, our case showed predominantly diffuse sheets of short spindle cells and nests of epithelioid cells in the primary tumor. Immunohistochemically, the tumor cells were positive for epithelial markers (AE1/AE3, EMA), a smooth muscle marker (desmin), and less specific sex-cord markers (CD56, WT-1, CD99), but negative for relatively specific sex-cord markers (α-inhibin, calretinin, FOXL2, and SF1). Although without well-formed tubules or retiform structure, the extensive sex-cord-like patterns in our case, including nests and trabeculae/cords, strongly suggested the diagnosis of UTROSCT rather than other mesenchymal tumors, such as LG-ESS with sex-cord differentiation. The details of five cases of *GREB1*-rearranged UTROSCT reported previously [1] contrasted with the cases described by Cheng-Han et al. and with the current case, because all five cases in that series had prominent sex-cord differentiation and were positive for α-inhibin and/or calretinin immunostaining. Whether *GREB1*-rearranged UTROSCT had specific morphological and immunohistochemical features should be investigated further when additional cases have been reported.

Regarding prognosis, *GREB1*-rearranged tumors tended to occur in significantly older women than did UTROSCT with *ESR1* fusions (51–70 years old, median: 65 years versus 34–55 years, median 47 years) [4]; moreover, *GREB1*-rearranged tumors tended to be larger and more mitotically active (0–14 versus 0–1) and appeared to behave more aggressively. The current case was a 57-year-old postmenopausal woman with a 10.0-cm uterine mass and three mitotic figures per 10 high-powered fields in the primary and recurrent tumors. The tumor cells were determined by FISH to have *NCOA2* rearrangement and by RNA sequencing to harbor a *GREB1-NCOA2* fusion. These pathological and clinical features of our case were consistent with the cases described by Lee et al. and provided evidence for the recurrent aggressive behaviors of *GREB1*-rearranged uterine tumors.

Seven cases of *GREB1*-rearranged uterine tumors were previously summarized [4]; of these, six cases had follow-up information, and two cases developed pelvic dissemination and/or lung metastasis with more than 1 year of follow-up. A recent report [1] described 26 cases of UTROSCT with *NCOA1–3* rearrangement. Of these, five cases were *GREB1*-rearranged UTROSCT. The only recurrent case in that report harbored *GREB1-NCOA2* fusion. The current case is the fourth known recurrent case with *GREB1* rearrangement in the literature. The tumor recurred 30 months after a total hysterectomy with bilateral salpingo-oophorectomy.

To date, 12 cases of *GREB1*-rearranged uterine sarcoma have been reported in the English literature [1–4, 19]; the current case is the 13th known case. The clinicopathological features of all known *GREB1*-rearranged tumors, including our case, are summarized in Table 1. The mean age of the patients was 64.8 years (range, 51–74 years). Follow-up information was available for nine cases (69.3%, 9/13), with a mean follow-up interval of 23.7 months (range, 0.75–66 months). Of these nine cases, four cases were recurrent or metastasized cases (44.4%). Of these, three tumors recurred in the pelvis (at 17, 30, and 66 months) and one metastasized to the lung after the initial diagnosis (at 24 and 132 months). The other five patients were alive without evidence of disease at the last follow-up (range, 0.75–54.3 months). The average tumor size was 9.1 cm (range, 4.2–14.9 cm). Microscopically, the tumor growth pattern of most tumors (10/12, 83.3%) was infiltrative growth (including three focal invasions; cases 2, 3, and 4), and two were well demarcated. Mitotic figures ranged from 0 to 14 per 10 high-powered field (2 mm^2^; mean: 3.6). Lymphovascular invasion and necrosis were each present in two cases (2/12, 16.7% and 2/7, 28.6%, respectively).

**Table 1:** Clinicopathologic features of GREB1-rearranged uterine sarcoma

Case number	Age (years)	Tumor size (cm)	Microscopic tumor margins	Tumor architecture	Cytomorphology	Mitosis (per 10 HPF)	LVI^**c**^	Necrosis	Fusion gene	Pathology diagnosis	Stage	Status of the disease
Cheng-Han et al. (2019)												
1	56	10.0	Well demarcated	Diffuse, fascicular, trabecular/corded	Epithelioid and spindle, occasional rhabdoid features.	8	–	–	GREB1-NCOA2	UUS-U^b^	IB	NED^d^ at 0.75 mo
2	60	14.9	Generally well-defined; rare foci of tongue-like protrusions	Diffuse, trabecular/corded, fascicular	Epithelioid and spindle	14	+	+	GREB1-NR4A3	GREB1-regrranged sarcoma	IB	NED at 5 mo
3	68	8.5	Myometrial delicate cell cords invasion	Trabecular/corded, diffuse, nested	Epithelioid	7	–	–	GREB1-SS18	Atypical mesenchymal tumor	IB	NED at 6 mo
4	65	4.2	Rare foci tongue-like protrusion	Trabecular/corded, diffuse, nested, fascicular; multifocal tubular/retiform structures	Epithelioid and spindle, rare lipid-laden cells and Leydig-like cells	2	–	–	GREB1-NCOA1	GREB1-regrranged sarcoma	IA	Recent case
Croce et al. (2019)												
5	70	10.0	Relatively well demarcated	Diffuse, nested, trabecular	Epithelioid, focal rhabdoid appearance	1	–	NA	GREB1-CTNNB1	UTROSCT	IB	Pelvic dissemination (17 mo); lung metastasis (30 mo)
Brunetti et al. (2018)												
6	51	6.5	Infiltrative	Primary tumor: diffuse fascicular; recurrent tumor: solid growth pattern	Primary tumor: spindle and polygonal cells displaying pronounced atypia; Recurrent tumor: predominantly epithelioid	multiple mitotic figures^a^	+	+	GREB1-NCOA2	High-grade endometrial sarcoma/sarcoma, not otherwise classifiable	IB	lung metastasis (24 and 132 y)
Dickson et al. (2019)												
7	68	0.7–3.3^a^	NA	Fascicles, focal tubular pattern	Spindle and epithelioid	0–1	NA	–	GREB1-NCOA2	UTROSCT	I	NA
Goebel et al. (2019)												
8	71	6.2	Infiltrative	Nested, corded	Spindle and epithelioid	1	–	NA	GREB1-NCOA1	UTROSCT	NA	NED at 54.3 mo
9	71	NA	Infiltrative	Sertoliform, retiform	Spindle and epithelioid	< 1	–	NA	GREB1-NCOA1	UTROSCT	NA	NA
10	71	13.0	Infiltrative	Corded, trabecular, sertoliform, retiform	Spindle and epithelioid	1	–	**NA**	GREB1-NCOA2	UTROSCT	NA	Recurrent to pelvis (66 mo)
11	74	at least 2.5	Infiltrative	Sertoliform	Spindle and epithelioid	3	–	NA	GREB1-NCOA1	UTROSCT	NA	NA
12	61	8.0	Infiltrative	Nested, corded, whorled	Spindle and epithelioid	2	–	NA	GREB1-NCOA1	UTROSCT	NA	NED at 10 mo
Present case												
13	57	10.0	Infiltrative	Diffuse, nested, trabecular/corded, and focal anastomotic glandular	Spindle and epithelioid	3	–	–	GREB1-NCOA2	UTROSCT	IB	Recurrent to pelvis (30 mo)

^a^Exact data not provided

^b^*UUS-U* undifferentiated uterine sarcoma with nuclear uniformity

^c^*LVI* lymphovascular invasion

^d^*NED* no evidence of disease

Of these 13 cases of *GREB1*-rearranged uterine tumors, eight cases (including our case) had a definite pathological diagnosis of UTROSCT, with typical morphological and/or immunohistochemical features. Five patients were initially diagnosed as having undifferentiated uterine sarcoma with nuclear uniformity, GREB1-rearranged sarcoma, atypical mesenchymal tumor, and sarcoma, not otherwise classifiable (cases 1, 2, 3, 4, and 6, respectively) (Table 1). Cheng-Han reported four cases (cases 1, 2, 3, and 4) as “GREB1-rearranged uterine sarcomas with variable sex-cord differentiation” because of limited sex cord differentiation and more aggressive clinical behavior. Notably, of these five cases, three cases (cases 2, 3, and 4) showed a focal trabeculae/cord pattern, which is an important morphologic clue for UTROSCT diagnosis. Except the four cases reported by Cheng-Han et al., seven patients with GREB1-NCOA2/NCOA1 fusion genes were diagnosed as having UTROSCT by authors (cases 5, 8, 9, 10,11, and 12), which suggested that at least a considerable proportion of these tumors with GREB1-rearrangement showed typical morphologic features of “UTROSCT”. Recently, three cases of UTROSCT with extensive rhabdoid differentiation, malignant behavior, and ESR1-NCOA2 fusions have been reported [20]. Whether these tumors with specific gene rearrangement and aggressive clinical behavior should be regarded as poorly differentiated UTROSCT or as distinct uterine sarcomas need to be further investigated when additional cases have been reported. However, two cases (cases 1 and 6) showed morphologic features of high-grade sarcoma, predominantly composed of diffuse sheets of epithelioid, spindle, or polygonal cells and without conspicuous sex-cord–like patterns or the sex-cord-like differentiation immunophenotype. This could indicate that molecular detection, including the *GREB1* rearrangement, should be used with cases of HG-ESS, undifferentiated sarcoma, and other high-grade sarcomas, which cannot be classified as any specific uterine sarcoma subtype, to clarify whether they are poorly differentiated UTROSCT or a distinct sarcoma subtype.

A systematic review by Blake et al. and a large case series by Moore et al. reported *GREB1*-rearranged uterine tumor recurrence rates of 5.9 and 23.5%, respectively; however, neither of these reports addressed the genetic findings [5, 6]. According to Moore et al., cases of UTROSCT that behaved in a malignant manner were associated with older age, larger tumor size, and higher mitotic activity [6]. These aggressive and/or older patients included in the study by Moore et al. may have had a high frequency of *GREB1* rearrangement or ESR1-NCOA2 fusions; however, the study did not address the molecular events. Additionally, the relatively higher recurrence rate in the series by Moore et al. may in part reflect a referral bias related to consultation cases in their series, which more often presented with unusual clinical behavior. Of these recurrent GREB1-rearranged cases, 3/4 harbored the GREB1-NCOA2 fusions and 1/4 the GREB1-CTNNB1 fusions. Interestingly, of the 4 UTROSCT cases harboring ESR1-NCOA2 with follow up information, three cases showed recurrence, as reported by Jennifer A. Bennett et al. [1, 20]. In contrast, no cases showed recurrence among the 10 cases of ESR1-NCOA3 positive UTROSCT with follow up information [1]. These limited recurrent cases specify that uterine tumor/sarcoma with GREB1-rearrangement or ESR1-NCOA2 fusions may have a high risk of recurrence/metastasis. Although the case number is limited, the higher recurrence rate of *GREB1*-rearranged or ESR1-NCOA2 fusion uterine tumors could indicate that these types have a greater tendency of aggressive behavior. Additional cases are needed to clarify the correlation between morphologic features, prognosis, and intrinsic molecular events.

Due to the rarity of this type of tumor, there is no well-established treatment protocol for UTROSCT, as there are limited data available for guiding clinical management. The initial clinical treatment strategies for UTROSCT include tumor resection, total abdominal hysterectomy alone, or total abdominal hysterectomy with bilateral adnexectomy, in consideration of patient age and parity. Recurrent cases are managed by repeated surgical procedures, and no evidence supports the usefulness of chemotherapy for recurrent cases [6]. Additional investigation of the correlation between specific molecular events and prognostic significance is essential for the development of therapeutic strategies, especially for tumors with aggressive behavior.

*GREB1* encodes growth regulation by estrogen in breast cancer 1, a protein transcriptionally driven by estrogen-bound ER. Furthermore, GREB1 is a key factor of the canonical estrogen/ER signaling pathway [21]. Functionally, GREB1 is one of the most important tamoxifen/RU486-suppressed ER pathway downstream effectors, which indicates that *GREB-*rearranged tumors may respond to tamoxifen and RU486 [4, 22]. *ESR1* encodes estrogen receptor 1, a ligand-dependent transcription factor. Binding with estrogen, ESR1 is not only essential for sexual development, reproductive function, and bone formation but is also involved in pathologic processes, including breast cancer, endometrial cancer, and osteoporosis. Several mutations in the ligand-binding domain of ESR1 have been found to be correlated with resistance to hormone therapy in ER-positive breast cancer [23, 24]. *ESR1*-rearranged UTROSCT may not be susceptible to an estrogen blockade because the ER ligand-binding domain is lost in fusions involving *ESR1* (4). The partner genes of *GREB1-* or *ESR1-*rearranged UTROSCT, including *NCOA1–3*, *NR4A3*, *SS18*, and *CTNNB1*, all encode transcription factors, and the basic function of each, as reported previously [4], is listed in Table 2. Five cases of UTROSCT that are suspected to be related to tamoxifen treatment have been reported [25]; however, the molecular features of these tamoxifen treatment-related cases were not determined. The complicated mechanism underlying chromosome translocation involving different functional genes, prognosis, and response to tamoxifen is still unclear. Although hormonal treatment has not yet been explored for UTROSCT, such in vitro and in vivo research may be necessary.

**Table 2:** Function of partner genes related to GREB1 rearrangement

Gene	Encoded protein	Basic function
*CTNNB1*	β-Catenin	Crucial transcriptional factor in Wingless-Int (Wnt)/β-catenin signaling pathway; transcriptional coactivator for T-cell factor/lymphoid enhancer factor (TCF), and with its transactivation domain, activates transcription initiation, histone methyltransferases, chromatin modification, and transcription facilitation
*NCOA1–3*	Nuclear Receptor Coactivator 1–3	Transcription factors: coactivate nuclear hormone receptors and mediate steroid/sex-hormone receptor pathways
*NR4A3*	Nuclear Receptor Subfamily 4 Group A Member 3	Transcription factor: transcriptional activator of the steroid/thyroid hormone nuclear receptor family, with a role in regulating proliferation, survival, and differentiation
*SS18*	SS18 (or SSXT)	Transcription factor: transcriptional coactivator and a component of the SWI/SNF chromatin-remodeling complex

In conclusion, we reported a case of recurrent UTROSCT in a 57-year-old woman with the *GREB1-NCOA2* fusion gene. This case provides further evidence that uterine tumors with *GREB1-*rearrangement may have a high recurrence/metastasis risk. Further studies are necessary to elucidate the clinical features of UTROSCT, especially the prognosis, potential treatments, and the range of possible molecular events.

## Data Availability

Not applicable.

## Abbreviations

UTROSCT: Uterine tumors resembling ovarian sex-cord tumor
ESR1: Estrogen receptor 1
NCOA: Nuclear receptor coactivator
GREB1: Growth regulation by estrogen in breast cancer 1
CTNNB1: Catenin beta 1
NR4A3: Nuclear receptor subfamily 4 group A member 3
SS18: Synovial sarcoma translocation, chromosome 18
ER: Estrogen receptor
PR: Progesterone receptor
WT-1: Wilm’s tumor-1
EMA: Epithelial membrane antigen
SF1: Stromal factor 1
FISH: Fluorescence in situ hybridization
LG-ESS: Low-grade endometrial stromal sarcoma
HG-ESS: High-grade endometrial stromal sarcoma
